# Malpractice

**Published:** 1854-02

**Authors:** 


*Malpractice.*—A suit was recently brought against Talbot Watts, the proprietor of a medicine known as "Watts' Nervous Antidote," by the mother of a girl 27 years of age, said to be afflicted with epilepsy, to whom Watts administered his medicine, with the promise that he would return the money if it failed to cure, which he afterwards declined doing. The medicine is said to have increased the frequency of the fits, and to have endangered her life.

The judge charged the jury among other things, that the same degree of skill ought not to be expected, and is not in law required in cases like this, as in cases where regular physicians are called in. Persons who take medicines from advertisements in newspapers, must, to a considerable extent, take the consequences.

The jury retired, and after deliberating about two hours, rendered a verdict for the plaintiff for *one thousand one hundred dollars.*—*N. Y. Medical Times.*

